# The Cardiac Effects of COVID-19 on Young Competitive Athletes: Results from the Outcomes Registry for Cardiac Conditions in Athletes (ORCCA)

**DOI:** 10.3390/jcdd10020072

**Published:** 2023-02-06

**Authors:** Nathaniel Moulson, Bradley J. Petek, Aaron L. Baggish, Kimberly G. Harmon, Stephanie A. Kliethermes, Manesh R. Patel, Timothy W. Churchill, Jonathan A. Drezner

**Affiliations:** 1 Division of Cardiology and Sports Cardiology BC, University of British Columbia, Vancouver, BC V6T2B5, Canada; 2 Massachusetts General Hospital Division of Cardiology, Boston, MA 02114, USA; 3 Cardiovascular Performance Program, Boston, MA 02114, USA; 4 Department of Cardiology, Lausanne University Hospital (CHUV), CH-105 Lausanne, Switzerland; 5 Institute for Sport Science, University of Lausanne (ISSUL), CH-105 Lausanne, Switzerland; 6 Department of Family Medicine and Center for Sports Cardiology, University of Washington, Seattle, WA 98195, USA; 7 Department of Orthopedics and Rehabilitation, University of Wisconsin Madison, Madison, WI 53705, USA; 8 Division of Cardiology, Duke Heart Center, Duke Clinical Research Institute, Duke University School of Medicine, Durham, NC 27710, USA

**Keywords:** athletes, COVID-19, SARS-CoV-2, myocarditis, sudden cardiac death, cardiovascular outcomes, preparticipation screening

## Abstract

The Outcomes Registry for Cardiac Conditions in Athletes (ORCCA) study is a large-scale prospective investigation evaluating the cardiovascular effects and outcomes of SARS-CoV-2 infection on young competitive athletes. This review provides an overview of the key results from the ORCCA study. Results from the ORCCA study have provided important insights into the clinical impact of SARS-CoV-2 infection on the cardiovascular health of young competitive athletes and informed contemporary screening and return to sport practices. Key results include defining a low prevalence of both cardiac involvement and adverse cardiovascular outcomes after SARS-CoV-2 infection and evaluating the utility of a return-to-play cardiac evaluation. Future aims of the ORCCA study include the longer-term evaluation of cardiovascular outcomes among athletes post-SARS-CoV-2 infection and the transition to investigating outcomes in young athletes with potentially high-risk genetic or structural cardiac diagnoses.

## 1. Introduction

The presence of cardiac involvement among patients hospitalized with severe COVID-19 was reported in the early stage of the COVID-19 pandemic [1,2,3]. This cardiac involvement was variably defined and associated with a multitude of cardiovascular complications. Electrocardiographic, cardiac biomarker, and cardiac imaging abnormalities and associations with myocardial injury, thrombosis, and arrhythmia have all been reported and associated with adverse outcomes among hospitalized patients [1,2,3]. The concern and uncertainty regarding the risk of cardiac involvement in young competitive athletes resulted in the development of multiple expert consensus recommendations for the cardiac evaluation of athletes after SARS-CoV-2 infection prior to their return to competitive athletics and training [4,5,6,7,8]. This concern arose not only from the findings in nonathlete hospitalized populations, but also from prior evidence identifying myocarditis as an important cause of sudden cardiac arrest and death (SCA/D) in young athletes [9,10,11].

Initial return-to-play (RTP) recommendations included a range of cardiac testing aimed at detecting subclinical SARS-CoV-2 cardiac disease [4,5,6,7]. Recommended cardiac tests included a resting 12-lead electrocardiogram (ECG), cardiac troponin (cTn), transthoracic echocardiography (TTE), and cardiac magnetic resonance imaging (CMR) when deemed appropriate. Most initial guidelines recommended “triad” testing, inclusive of an ECG, cTn, and TTE among athletes with mild or greater symptoms [4,5,6,7]. Upfront or screening CMR was recommended by several institutions due to concern around the limited sensitivity of other modalities to detect subclinical disease, and from preliminary reports identifying high rates of CMR abnormalities in nonathlete populations [12,13,14]. These initial recommendations were based on expert opinion and emerging clinical experience but lacked support from scientific data [4,5,6,7]. 

**Figure 1:**
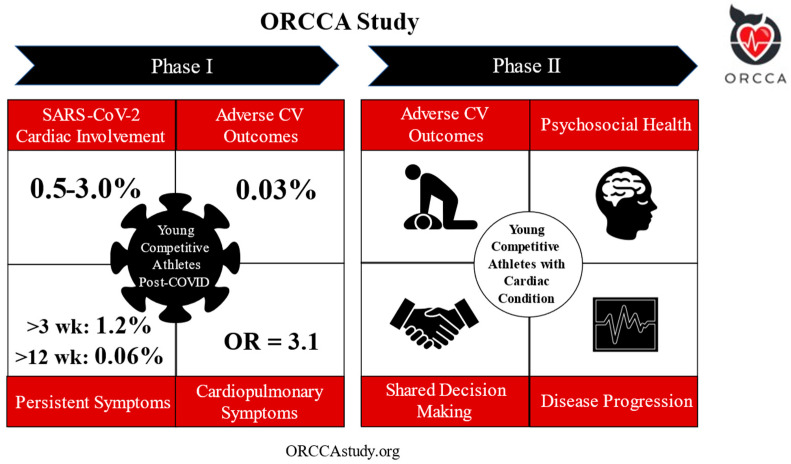
Overview of ORCCA Phase 1 and Phase 2. Reproduced from ACC.org [16]. Definition of abbreviations: OR = odds ratio. For Phase 1, the prevalence of SARS-CoV-2 cardiac involvement was found to be 0.5–3.0% [15]. On a median follow-up of >1 year (*n* = 3675), there was one adverse event (0.03%) possibly related to SARS-CoV-2 infection [17]. The prevalence of persistent symptoms following SARS-CoV-2 infection >3 weeks was found to be 1.2%, and >12 weeks in 0.06% of athletes [18]. Cardiopulmonary symptoms were associated with SARS-CoV-2 cardiac involvement in multivariate analysis with odds ratio 3.1 [95% CI 1.2–7.7] [15]. Phase 2 focusses on “high-risk” cardiac conditions and aims to monitor for (1) cardiovascular outcomes; (2) the process and outcome of competitive sport eligibility decisions; (3) physical activity and exercise habits after diagnosis; (4) psychological impact, mental health, and quality of life of living with a cardiovascular condition; (5) rate and magnitude of disease progression; and (6) changes to or implementation of a specific emergency action plan at the athlete’s institution (see Future Aims section below).

The Outcomes Registry for Cardiac Conditions in Athletes (ORCCA) was established to address these concerns. This prospective study enrolled collegiate athletes from the National Collegiate Athletic Association (NCAA) in the United States with confirmed SARS-CoV-2 infection. The study collected data documenting initial SARS-CoV-2 illness course and severity, cardiac testing results, cardiac and noncardiac diagnoses, and attendant clinical outcomes for athletes returning to organized competitive athletics following infection [15]. Several key practice informing results have been published from this work that included enrollment of 3675 athletes from 45 separate institutions and over one year of published cardiovascular outcomes follow-up (September 2020 to November 2021) (Figure 1) [16].

This review provides an overview of the key results from the ORCCA study with comparison to other large observational studies assessing the effect of COVID-19 on young competitive athletes. We also introduce current ORCCA study aims [16].

## 2. Clinical Presentations of COVID-19 Infections in Young Athletes 

### 2.1. Acute COVID-19 Infection

Acute COVID-19 infection resulted in the development of symptomatic infection in 67% of athletes within the initial ORCCA study [15]. Asymptomatic infection diagnosed through containment screening strategies including surveillance testing, return-to-campus testing, and contact tracing, accounted for 33% of COVID-19 positive athletes. In symptomatic athletes, illness severity was mild in 29%, and moderate in 25%. Importantly, 13% of athletes experienced acute cardiopulmonary symptoms including chest pain, shortness of breath, palpitations, presyncope/syncope, or exercise intolerance. Hospitalization in the ORCCA cohort was rare (0.2%) with acute infection and entirely related to noncardiac complications. No athlete required the intensive care unit. 

### 2.2. Persistent Symptoms following COVID-19 Infection 

Post-acute sequalae of COVID-19 (PASC) defines the constellation of new, returning, or persistent symptoms or health problems occurring after SARS-CoV-2 infection [18]. There is a wide range of symptoms commonly experienced including exercise intolerance, fatigue, cognitive dysfunction (“brain fog”), and sleep disturbance, but effects on nearly every organ system have been reported [18]. Contemporary definitions now define this entity as occurring 4 or more weeks after initial infection; however, the time course of this definition has evolved over the pandemic. The ORCCA study evaluated close to 3600 athletes for the presence of persistent symptoms after COVID-19 infection and found that persistent symptoms were relatively infrequent in the young competitive athlete population [19]. Only 1.2% of athletes reported symptoms greater than 3 weeks, 0.8% greater than 4 weeks, and 0.06% reported symptoms greater than 12 weeks. Of those athletes with persistent symptoms, the predominant symptom reported was loss of taste and/or smell (63%), followed by shortness of breath (20%), cough (15%), chest pain (15%), and fatigue (10%).

### 2.3. Importance of Exertional Cardiopulmonary Symptoms 

Exertional cardiopulmonary symptoms are defined as the presence of chest pain, shortness of breath, palpitations/tachycardia, presyncope/syncope, or exercise intolerance/fatigue on return to exercise [19]. Exertional cardiopulmonary symptoms were observed in 4.0% of athletes upon return to exercise or sport in the ORCCA study. Clinical evaluation and diagnostic testing led to a diagnosis of SARS-CoV-2 associated sequelae in almost 1 in 10 athletes with exertional cardiopulmonary symptoms. Importantly, this included five athletes with probable or definite SARS-CoV-2 cardiac involvement, and all five athletes experienced chest pain on return to exercise. Other diagnoses included pneumonia, inappropriate sinus tachycardia, postural orthostatic tachycardia syndrome (POTS), and a large pleural effusion. These findings highlight the importance of continued monitoring during the return-to-play period, even in those with asymptomatic or mild acute illnesses, and the need for a comprehensive evaluation in athletes who develop exertional cardiopulmonary symptoms, especially those with chest pain upon return to exercise [18,19].

## 3. SARS-CoV-2 Cardiac Involvement in Young Athletes

Inflammatory heart disease such as myocarditis and pericarditis have well-established clinical diagnostic criteria for the general population [20,21]. However, these definitions are predicated on the presence of a clinical presentation suggestive of disease. Precise definitions for subclinical myocarditis in the cardiovascular screening context were not clearly defined prior to the COVID-19 pandemic onset and remain an area of diagnostic and clinical uncertainty. Initial single-center observational cohort studies of athletes undergoing RTP cardiac testing inclusive of CMR reported highly variable rates of SARS-CoV-2 cardiac involvement (1.4 to 56%) in part due to the utilization of heterogeneous disease definitions and the potential for variable interpretations between institutions [13,22,23,24].

The ORCCA study investigators derived disease definitions for both SARS-CoV-2 myocardial and pericardial involvement along a spectrum of certainty (definite, probable, possible) with imaging components adapted from the modified Lake Louise imaging criteria [15,25]. This adaptation was necessary to convey a degree of certainty for the diagnosis, as the modified Lake Louise criteria were not validated as a screening tool for low-to-intermediate risk populations with associated symptomatology [25]. Cardiac involvement was defined as myocardial, pericardial, or both, and by the degree of certainty for the diagnosis (definite, probable, possible). Definite and probable involvement reflect the higher degree of certainty for the presence of cardiac involvement. Possible involvement reflects a finding of uncertain clinical or prognostic significance and was not subsequently considered a relevant sequela of SARS-CoV-2 within the ORCCA data [17,19].

The primary outcome of the initial publication from the ORCCA study evaluated the prevalence of SARS-CoV-2 cardiac involvement in a cohort of 3018 athletes (mean age, 20 years (SD, 1 year); 32% female) undergoing RTP cardiac evaluation. This cohort consisted of 198 athletes in whom primary screening CMR was performed and the remainder (*n* = 2820) who underwent at least one element of triad testing followed by CMR only if clinically indicated. The overall prevalence of definite, probable, or possible SARS-CoV-2 cardiac involvement was 0.7%. In the population of athletes undergoing clinically indicated CMR, the prevalence of cardiac involvement was 0.5%. In athletes undergoing a primary screening CMR, the prevalence of cardiac involvement was 3.0%. However, half of the diagnoses in athletes undergoing primary screening CMR were deemed “possible” involvement of uncertain clinical significance. 

The prevalence estimate reported from ORCCA is similar to other published registry data from professional and collegiate athletes undergoing RTP cardiac screening [14,26]. The prevalence of cardiac involvement was 0.6% among 789 US professional athletes undergoing a protocol of triad testing followed by CMR when clinically indicated [26]. In a separate cohort of 1597 US collegiate athletes undergoing a protocol of primary screening CMR, 2.3% were noted to have imaging evidence of cardiac involvement. However, only 0.6% (*n* = 9) of athletes had findings consistent with clinical myocarditis [14]. The ORCCA study remains the largest study to date assessing the prevalence of SARS-CoV-2 cardiac involvement in young competitive athletes [27].

## 4. Cardiovascular Outcomes in Athletes following COVID-19 

In addition to defining the prevalence of SARS-CoV-2 cardiac involvement, the ORCCA study prospectively evaluated the rate of adverse cardiovascular outcomes after infection [15,17]. A total of 3675 athletes were followed for a median of 1.12 years (IQR, 1.06, 1.22). The risk of clinically relevant adverse cardiovascular outcomes was low with two observed adverse cardiac events (0.05%). Both adverse cardiac outcomes occurred in athletes without SARS-CoV-2 cardiac involvement. One athlete had successfully resuscitated sudden cardiac arrest attributed to preexisting genetic structural heart disease as adjudicated by a multi-institutional panel, and a second athlete developed new onset atrial fibrillation occurring <2 weeks after SARS-CoV-2 infection but with no cardiac involvement noted on CMR. This athlete underwent a successful electrical cardioversion without reported occurrence over the follow-up period. 

This study also evaluated the specific population of athletes who were diagnosed with SARS-CoV-2 cardiac involvement (*n* = 21) providing insight to their clinical course. All of these athletes were initially restricted from sport for a median of almost 3 months (86 days). Twenty athletes successfully returned to competitive sport, and one athlete chose not to return despite medical clearance. Repeat CMR was performed in 10/21 (48%) athletes. Seven athletes had complete resolution of prior imaging abnormalities, one athlete had partial resolution (resolution of T2 abnormality with persistent late gadolinium enhancement (LGE)), and two athletes had persistent imaging abnormalities (persistent T2 and persistent T1/LGE). After a median follow-up of 1.12 years (IQR 1.06, 1.22), no adverse cardiovascular outcomes were reported among this athlete cohort. 

## 5. Post-SARS-CoV-2 Infection Return-to-Play Cardiovascular Evaluation

### 5.1. Contemporary Approach to Return-to-Play Screening 

The 2022 American College of Cardiology (ACC) Expert Consensus Decision Pathway on Cardiovascular Sequelae of COVID-19 in Adults now recommends a symptoms-based approach to assess the need for cardiac testing in athletes returning to competitive sport or intense training after infection [18]. This approach recommends cardiac evaluation and testing only among athletes with cardiopulmonary symptoms as part of their acute illness, or if new cardiopulmonary symptoms develop on RTP. These recommendations were heavily informed by the results of the ORCCA study publications [15,17,19]. 

A symptoms-based approach serves to reduce the estimated costs and healthcare resource utilization associated with universal screening. The cost of universal screening with ECG, biomarkers, and TTE has been estimated to be USD 1072.30 ± 517.93 per athlete [28].

Since publication of this statement, several additional key insights from the ORCCA study have been reported [29,30,31,32]. This work has focused on defining the utility and quality of specific components of cardiac testing (ECG, cTn, TTE) utilized for screening young athletes for COVID-19 related and non-COVID cardiac pathology. 

### 5.2. History 

**Figure 2:**
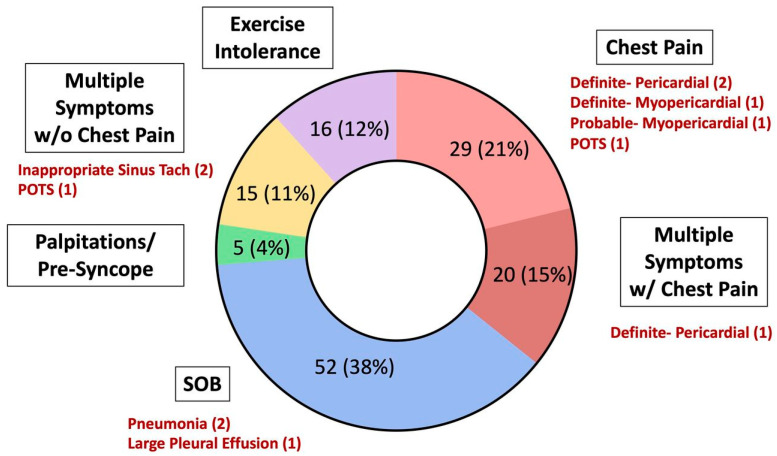
Reproduced from Petek et al. Open Access 2022, *British Journal of Sports Medicine* [19]. SARS-CoV-2-associated clinical sequelae in athletes with exertional cardiopulmonary symptoms on return to exercise stratified by symptom type. POTS: postural orthostatic tachycardia syndrome; SOB: shortness of breath; w/: with; w/o: without.

As highlighted previously, the importance of cardiopulmonary symptoms in young athletes either during initial infection or on RTP is a key finding from the ORCCA study. The presence of cardiopulmonary symptoms is associated with an odds ratio of 3.1 for SARS-CoV-2 cardiac involvement [15]. Exertional chest pain appears to be of particular significance. In athletes with exertional cardiopulmonary symptoms on RTP, 21% of those with chest pain who underwent CMR were diagnosed with probable or definite SARS-CoV-2 cardiac involvement, while no athlete with exertional cardiopulmonary symptoms without chest pain was found to have cardiac involvement (Figure 2) [19]. Therefore, exertional chest pain on RTP is a concerning finding that should prompt subsequent cardiovascular testing as recommended by contemporary guidelines [18].

### 5.3. ECG 

Preparticipation cardiovascular screening of young competitive athletes with the use of ECG is used to improve detection of pathological cardiac conditions [33,34,35]. The results from the ORCCA study have raised several questions around the role of the ECG in the detection of subclinical SARS-CoV-2 cardiac involvement. First, only 1/21 (5%) athletes diagnosed with SARS-CoV-2 cardiac involvement from the ORCCA study demonstrated ECG abnormalities [15]. Second, in athletes with SARS-CoV-2 infection but without cardiac involvement, around 4% developed new ECG abnormalities of uncertain significance, and 50% of athletes with previously abnormal ECGs at baseline had normal ECGs following their COVID-19 infection [29]. These results further support a symptoms guided approach to RTP cardiac testing in athletes after SARS-CoV-2 infection. 

### 5.4. Troponin

**Figure 3:**
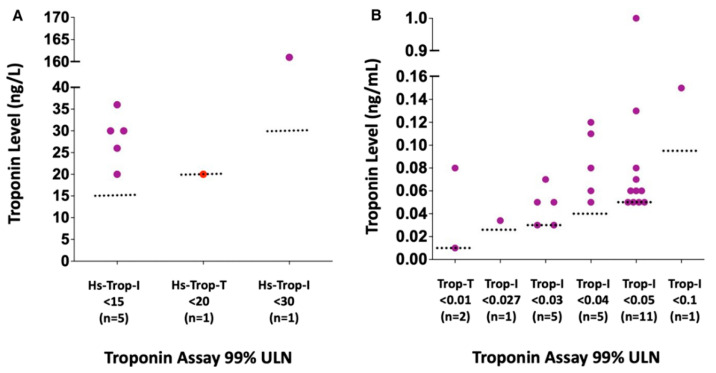
Magnitude of troponin elevation in athletes with abnormal troponin levels. Reproduced from Moulson et al. [32] Open Access, 2022, *Journal of American Heart Association*. (**A**) High-sensitivity troponin assays. (**B**) Traditional troponin assays. Red dots indicate probable or definite SARS-CoV-2 myocardial involvement. Purple dots = athletes with abnormal troponin but no evidence of probable or definite SARS-CoV-2 myocardial involvement; red dots = athletes with abnormal troponin and evidence of probable or definite SARS-CoV-2 myocardial involvement. Hs indicates high sensitivity. Trop: troponin; Trop-I: troponin-I; Trop-T: troponin-T; and ULN: upper limit of normal.

Prior to the COVID-19 pandemic, cTn testing had not been utilized in the cardiovascular screening context for young athlete populations. As elevations in cTn are often found in those with clinical myocarditis, and elevations in cTn were both frequent and of prognostic significance among hospitalized patients with COVID-19, cTn testing became an initial mainstay of RTP cardiac testing for athletes [3,6,7,21]. This practice was despite an absence of rigorous scientific data on the diagnostic performance of cTn utilized in this context. Among the 3184 athletes who underwent screening cTn in the ORCCA study (median of 13 days after COVID-19 infection), a total of 32 (1%) athletes were found to have an abnormal cTn level (defined as >99% upper limit of normal; Figure 3) [32]. Among these patients with abnormal cTn, a total of 19 underwent CMR and only 1/19 (5%) met criteria for definite or probable SARS-CoV-2 cardiac involvement. While several plausible explanations for this finding have been proposed, it has drawn into question the use of cTn as a screening tool in patients with a low pretest probability for myocarditis [32].

### 5.5. Echocardiography

The use of noninvasive imaging for preparticipation cardiovascular screening of young athletes has not been recommended outside of the context of the COVID-19 pandemic [36]. For the reasons outlined above, cardiac imaging, predominately in the form of TTE, was recommended as a component of initial screening in young athletes prior to RTP following COVID-19 infection [4,5,6,7]. Given the widespread use of TTE among young competitive athletes after SARS-CoV-2 infection, this provided a unique opportunity to evaluate real-world data on TTE screening relating to both SARS-CoV-2 cardiac involvement and non-COVID-19 cardiac pathology. 

One of the important findings from data collected during the ORCCA study was that many TTE protocols during the COVID-19 pandemic did not comprehensively evaluate for common causes of SCD among young competitive athletes [9,10]. Coronary anomalies represent an important cause of SCD in young athletes and are typically electrically silent at rest and thus not detected by preparticipation ECG screening [9,10]. Contemporary guidelines recommend assessment of the proximal coronary origins regardless of the indication for echocardiography [36], and this evaluation has been shown to be effective and feasible in multiple prior studies [37]. To evaluate the frequency of compliance with this recommendation, we reviewed 1529 TTE reports from 56 different imaging laboratories from the ORCCA study and classified each laboratory as a “consistent reporter” (defined as documenting the ability or inability to visualize the proximal coronary ostia in >90% of reports) versus “variable reporters” (reporting in <90% of cases). Notably, only 18% of echocardiography laboratories reported on coronary origins at all, and only 5/56 (9%) met the definition of “consistent reporters”. 

Aortic dissection is another important cause of SCD among young competitive athletes [9,10]. Congenital aortopathies are also electrically silent, so they often go undetected unless found by imaging. Taking a similar approach, we reviewed TTE reports from 1521 athletes and 56 distinct echocardiography laboratories from the ORCCA study, defining “consistent reporters” if the aortic root or ascending aorta was reported in ≥75% of TTE reports [31]. Reassuringly, all echocardiography laboratories consistently reported on the aortic root (100%), but only 48% met the defined “consistent reporter” criteria for the ascending aorta (38% when limited to laboratories submitting ≥5 cases). In aggregate, these data highlight the need for quality improvement among echocardiography laboratories, as imaging of the coronary artery origins and ascending aorta should be universal components of any TTE performed in a young competitive athlete [36].

### 5.6. CMR 

Data from the ORCCA study has been instrumental in informing contemporary expert consensus-based practice [18,38,39]. This approach recommends against the use of primary screening CMR. Primary screening CMR appears to increase the diagnosis of cardiac involvement [14,15]; however, the clinical significance of subclinical CMR abnormalities found in athletes without other cardiac testing abnormalities or symptoms suggestive of inflammatory heart disease remains uncertain [40]. The use of CMR should therefore be reserved for those with a clinical syndrome consistent with myocarditis. Guidelines currently recommend use of CMR in two situations: (1) among athletes with initial cardiopulmonary symptoms and abnormal cardiac triad testing, or (2) in athletes with persistent or new onset cardiopulmonary symptoms (i.e., chest pain on return to exercise) [18].

The heterogeneity in prevalence estimates of SARS-CoV-2 cardiac involvement between institutions has also shed light on the need for rigorous scientific evaluation of CMR in the detection of inflammatory heart disease in athletes [13,14,15,22,23,24]. In a study of 13 Big Ten schools, the prevalence of CMR-defined SARS-CoV-2 cardiac involvement ranged between 0–7.6% [14]. This heterogeneity is most likely explained by variations in site image acquisition and interpretation variability, and not true differences in the rates of inflammatory heart disease. Future work should aim to optimize image acquisition techniques, define normative values, and create educational tools for the interpretation of CMR in athletes.

## 6. Future Aims of the ORCCA Study

### 6.1. Cardiovascular Outcomes after SARS-CoV-2 Infection

A main limitation of the current published data on the cardiovascular effects of SARS-CoV-2 infection in young athletes is an absence of longer-term follow-up. The ORCCA study plans to provide additional follow-up data to evaluate for the possibility of adverse cardiac outcomes in young athletes with and without previously diagnosed cardiac involvement. 

### 6.2. Outcomes in Athletes with “High-Risk” Cardiac Conditions 

An absence of outcomes data also exists among competitive athletes diagnosed with a potentially high-risk cardiac conditions associated with SCA/D. Our contemporary understanding of the natural history and corollary outcomes of young competitive athletes with many forms of cardiovascular disease remains rudimentary [16]. The ORCCA study and established infrastructure has begun investigating the long-standing (pre-pandemic) areas of clinical uncertainty in the management and outcomes of young competitive athletes with a potentially high-risk cardiac diagnosis. Our primary aim is to monitor cardiovascular outcomes in competitive athletes with a condition at risk of SCA/D, including athletes that continue playing or withdraw from competitive sport (Figure 1). Key secondary outcomes include (1) the process and outcome of competitive sport eligibility decisions; (2) physical activity and exercise habits after diagnosis; (3) psychological impact, mental health, and quality of life of living with a cardiovascular condition; (4) rate and magnitude of disease progression; and (5) changes to or implementation of a specific emergency action plan at the athlete’s institution. This phase of the ORCCA study is actively enrolling through the utilization of an online portal (https://orccastudy.org; accessed on 15 December 2022). 

## 7. Conclusions

The ORCCA study has provided key insights into the effects of SARS-CoV-2 infection on the cardiovascular health of young competitive athletes. This includes defining the prevalence of cardiac involvement after infection, monitoring for adverse cardiovascular events, and evaluating the utility of RTP cardiac screening. The ORCCA study has informed contemporary recommendations for a symptoms based approach to the RTP cardiac evaluation in athletes with SARS-CoV-2 infection. Future aims of the ORCCA study include the long-term monitoring and outcomes among young competitive athletes diagnosed with traditional genetic, structural, and acquired cardiac conditions associated with SCA/D.
